# Extrafollicular Cystic Adenomatoid Odontogenic Tumor Misdiagnosed as Glandular Odontogenic Cyst

**DOI:** 10.30476/DENTJODS.2022.91410.1577

**Published:** 2022-09

**Authors:** Fatemeh Mashhadiabbas, Pedram Bakhshaei, Sanaz Gholami Toghchi, Roohollah Safarpour

**Affiliations:** 1 Dept. of Oral & Maxillofacial Pathology, Dental School, Shahid Beheshti University of Medical Sciences, Tehran, Iran; 2 Dept. of Oral & Maxillofacial Surgery, Dental School, Shahid Beheshti University of Medical Sciences, Tehran, Iran; 3 Postgraduate Student of Oral and Maxillofacial Pathology, Dental School, Shahid Beheshti University of Medical Sciences, Tehran, Iran

**Keywords:** Adenomatoid odontogenic tumor, Mandible, Benign, Cyst

## Abstract

Adenomatoid odontogenic tumor (AOT) is a benign slow-growing, asymptomatic epithelial odontogenic neoplasm. This lesion has been known for its varied clinical and histoarchitec-tural patterns. Most AOTs occur intra-osseously in the anterior maxilla associated with the unerupted tooth. Clinically, AOT is sometimes misdiagnosed as an odontogenic cyst. Alt-hough enucleation and curettage for AOT is the most common treatment modality, accurate histopathological diagnosis is essential to avoid unnecessary extensive surgery. Here, we present a rare challenging case of an extrafollicular cystic adenomatoid odontogenic tumor occurring in the body of the mandible in a 23-year-old female patient, which was diagnosed clinically and radiographically as glandular odontogenic cyst. The diagnosis of adenomatoid odontogenic tumor was confirmed through histopathological examination

## Introduction

The latest WHO classification of odontogenic tumors defines adenomatoid odontogenic tumor (AOT) as a proliferation of odontogenic epithelium exhibiting a variety of histo-architectural patterns embedded in mature connective tissue stroma characterized by slow and non-invasive growth [ 1 ]. AOT is a relatively rare epithelial odontogenic neoplasm representing 3% to 7% of all odontogenic tumors [ 2 ]. A total of 73% of cases demonstrate a well-circumscribed, unilocular radiolucency containing the crown of an unerupted tooth (follicular type) which mimic dentigerous cysts. The extrafollicular variant (24% of cases) is a well-defined, unilocular radiolucency not related to unerupted teeth, and is located between the roots of erupted teeth. The rare extraosseous type (3% of cases) that arises in the gingival tissue has also been reported [ 3 ]. Cystic and classic (solid) AOTs are categorized depending on the macroscopic (gross) appearance and histopathology, not from the radiological features [ 4 ]. The histopathological characteristics of this lesion are known and making its diagnosis easy and definite [ 2 ].

Here, we present an uncommon case of extrafollicular cystic AOT in female patient, in an unusual region of the mandible, which was diagnosed clinically and radiographically as glandular odontogenic cyst, while histop athologically was confirmed as AOT.

## Case Presentation

**Figure 1:**
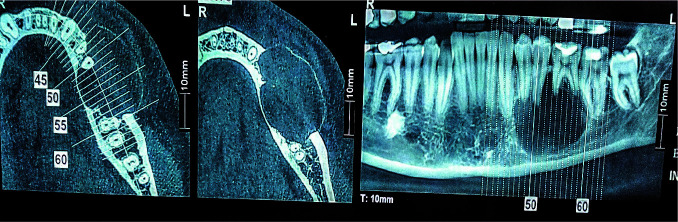
Cone beam computed tomography (CBCT) scans revealed a well-defined corticated unilocular radiolucent lesion of 34-37

**Figure 2:**
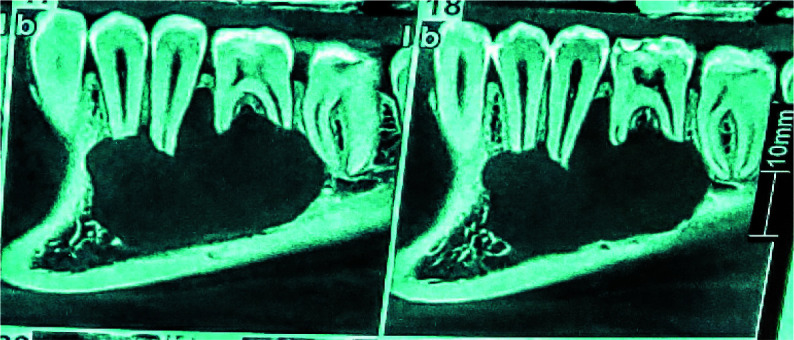
Scalloping unilocular radiolucent, root resorption of 34, 35, 36

**Figure 3:**
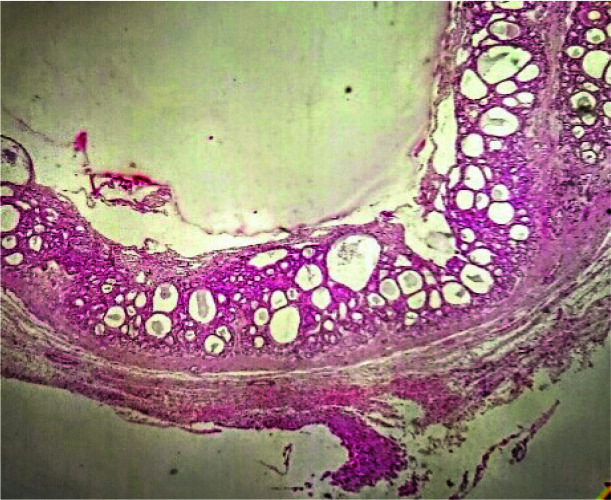
Low power histopathologic image demonstrating an encapsulated cystic lesion with papillary projections extending into the cyst lumen in some areas (40× Magnification, H&E)

**Figure 4:**
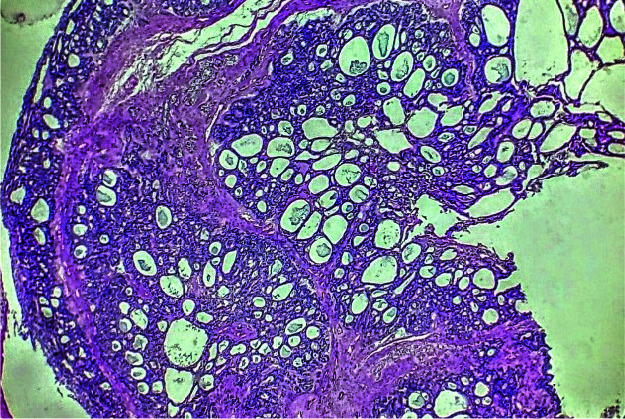
Cribriform pattern of epithelial cells (100 × Magnification, H&E)

**Figure 5:**
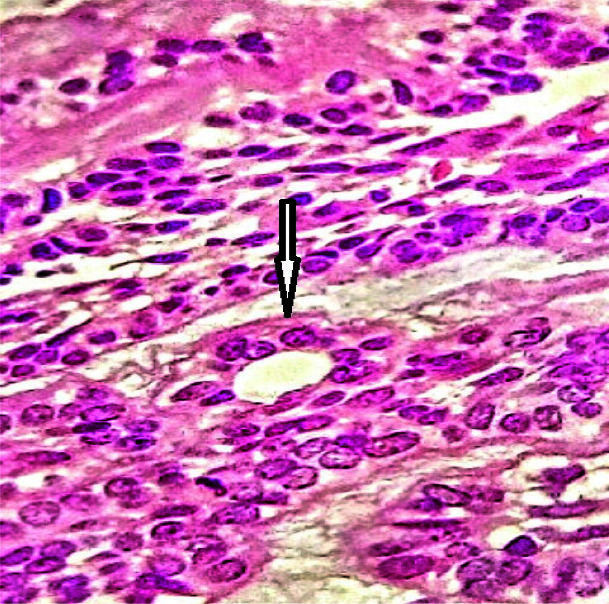
Duct-like structure, which is the characteristic feature of adenomatoid odontogenic tumor (arrow) (400× magnification, H&E)

**Figure 6:**
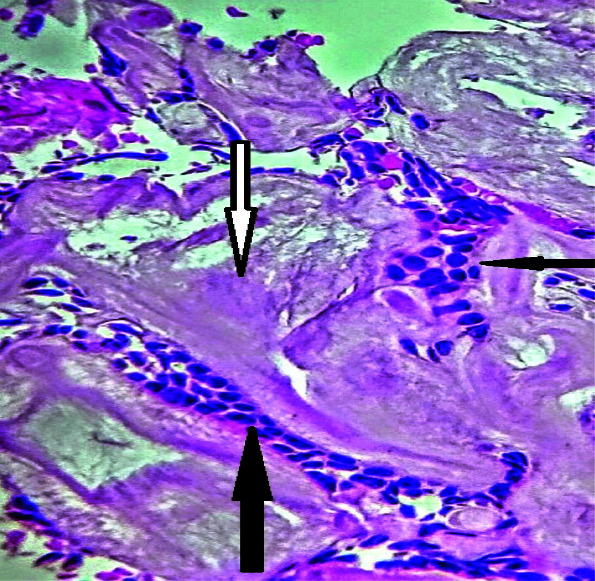
Eosinophilic/hyaline material (white arrow) which surrounded by odontogenic epithelium (black arrow) (400 ×Magnification, H&E)

**Figure 7:**
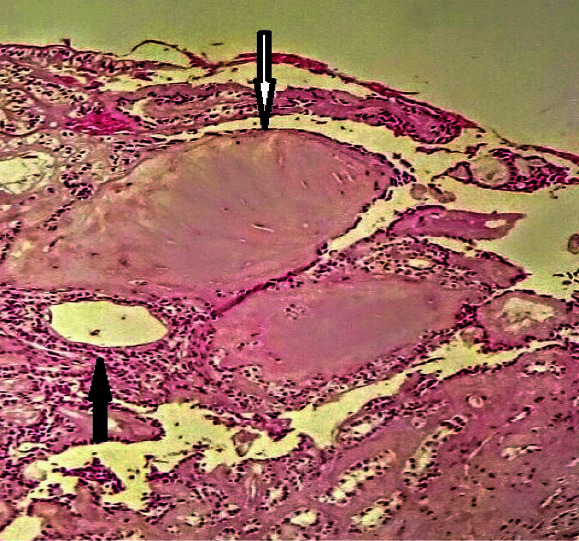
Dentinoid-like material (white arrow) adjacent the duct-like structure (black arrow) (100× Magnification, H&E)

**Figure 8:**
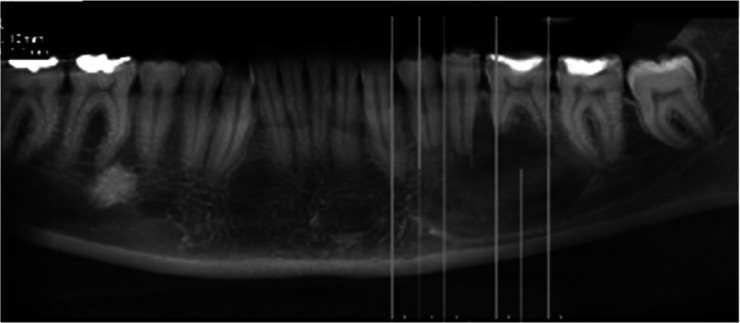
Post-operative cone beam computed tomography (CBCT) image shows normal healing and no signs of recurrence

A 23-year-old female was admitted to the Department of Oral and Maxillofacial Surgery of Shahid Beheshti Dental School (Tehran, Iran) for examination of a swelling of the left posterior mandible since 4 months ago. The patient had no history of previous trauma or medical problems. Her extraoral examination revealed mild swelling over the left posterior mandible. Intraoral examination revealed buccal cortical expansion in the area of left mandibular first premolar and the second molar, while the overlying mucosa was intact and pink in color. Cone beam computed tomography (CBCT) radiograph revealed a well-defined corticated unilocular radiolucent lesion between roots of 34-37, measuring about 2×3cm in size (Figure 1). Because of the lesion, the roots of the left mandibular first and second premolars plus first molar were resorbed (Figure 2). There was no cervical lymphadenopathy and blood investigation was within normal limits. Differential diagnosis based on radiographic features and vitality tests, included glandular odontogenic cyst, odontogenic keratocyst (OKC), and unicystic ameloblastoma. The lesion was completely excised under local anesthesia. The gross imitated a cystic lesion, and was a creamy-brown elastic tissue. The maximum wall thickness was 2mm. The histopathologic sections showed an encapsulated cystic benign epithelial odontogenic tumor composed of sheets, ducts, strands of cuboidal to spindle-shaped odontogenic epithelial as well as clear cells with cribriform, plexiform pattern and papillary projections into the lumen in some areas (Figures 3 and Figure 4). The duct-like structures were lined by cuboidal epithelial cells (Figure 5). Some amounts of eosinophilic/ hyaline materials with a few entrapped cells were seen surrounded by odontogenic epithelium (Figure 6). In some areas, dentinoid-like material was also evident (Figure 7). Correlating the histopathological features with the clinical and radiographic findings, a final diagnosis of an extrafollicular cystic AOT was made. The postoperative course was uneventful and there were no signs of recurrence until 8 months later (Figure 8). Informed consent was obtained from the patient for the surgical procedure and the necessary information for reporting this case. 

## Discussion

Despite the passage of more than a century following the initial report, the AOT remains a widely researched tumor due to its unique biological profile [ 4 ]. This tumor has a high tendency toward women in the second decade of life [ 5 ]. Here, we reported a female patient which was in her third decade of life. AOT has a striking tendency to occur in the anterior portion of the jaws (95%) and is found twice as often in the maxilla (65%) than in the mandible [ 6 ]. In the present case, the lesion occurred at an unusual site, posterior region of the mandible. AOTs are usually asymptomatic and are discovered during routine radiographic examinations. Large intraosseous lesions show painless expansion of the jaw [ 2 ], as in the case reported here. Root resorption, which is frequently presented in lesions such as ameloblastoma, is a very rare finding. Up to now, only 7 cases of AOT with root resorption have been reported [ 6 ]. However, root resorption of the teeth and not large lesion in our case are unusual in an AOT. Radiographically, most AOTs are well-demarcated with almost always unilocular radiolucency of smooth corticated and sometimes sclerotic borders. As in the present case, scalloped border and root resorption are rarely seen. However, divergence of adjacent roots and displacement of teeth may be observed [ 2 ]. Scalloped margins (13% of cases) on radiographs and root resorption (22%) in glandular odontogenic cyst [ 7 ] are the features, which led to early misdiagnosis of the present case. AOT has been known for its varied histo-architectural patterns [ 5 ]. AOT is usually surrounded by a thick, fibrous capsule. It is composed of spindle-shaped epithelial cells that form sheets, strands, whorled masses, rosette-like, or duct-like structures in a fibrous connective tissue. Abortive enamel formation, dentinoid material, and cementum may also be scattered throughout the neoplasm [ 2 , 5 ]. As many as 20 different histological patterns of AOT have been described in the literature, such as cystic variant of AOT, cribriform pattern, nests-like pattern, plexiform pattern, ribbon-like pattern, or clear cell changes [ 8 ]. 

Our case has been unique because of the varied AOT features seen in the cyst, grossly and histopathologically composed of cystic variant, cribriform pattern, plexiform pattern, luminal proliferations into the cystic lumen, sheets of tumor cells, and clear cell changes. AOT is rarely seen as completely cystic in the microscopic feature [ 5 ]. Marx *et al.* [ 9 ] considered AOT as a cyst and not a tumor and further gave a new terminology for this lesion as adenomatoid odontogenic cyst (AOC). Philipsen *et al.* [ 10 ] have strongly argued in favor of the concept of AOT being derived from the complex system of dental lamina or its remnants and preferred the name AOT rather than AOC. In classical AOT (solid), the proliferation of nodules originating from the cystic lining fills up the entire lumen while in cystic variant this process is incomplete; thus it is seen only in parts of cystic lining [ 4 ], as the present case with cystic and incomplete proliferation of nodules. AOT is often misdiagnosed as an odontogenic cyst [ 6 ], such as our case whose condition was misdiagnosed as glandular odontogenic cyst. Jayasooriya *et al.* [ 4 ] explained that cystic AOTs tend to present significantly larger than classic (solid) AOTs; they reported no significant clinico-pathological differences between cystic and classic AOTs. Thus, the cystic AOTs can be considered as a variant of AOT. The treatment of choice in all variants (AOT, AOC and extraosseous) is conservative surgery (enucleation) with no evidence of recurrence [ 5 ]. The cystic AOTs can be considered as a variant of AOT with enucleation, conservative excision, or radical excision as the treatment of choice depending on the extent of the lesion, similar to classic AOTs [ 4 ].

## Conclusion

AOT sometimes resembles an odontogenic cyst. Although enucleation and curettage for AOT constitute the most common treatment modality, accurate histopathological diagnosis is required to avoid unnecessary extensive surgery.

## Conflict of Interest

The authors declare that they have no conflict of interest.
